# Defining and quantifying the resilience of responses to disturbance: a conceptual and modelling approach from soil science

**DOI:** 10.1038/srep28426

**Published:** 2016-06-22

**Authors:** L. C. Todman, F. C. Fraser, R. Corstanje, L. K. Deeks, J. A. Harris, M. Pawlett, K. Ritz, A. P. Whitmore

**Affiliations:** 1 Rothamsted Research, Harpenden AL5 2JQ, UK; 2 Cranfield University, Cranfield, Bedford MK43 0AL, UK; 3 School of Biosciences, The University of Nottingham, Sutton Bonington Campus, Leicestershire LE12 5RD, UK

## Abstract

There are several conceptual definitions of resilience pertaining to environmental systems and, even if resilience is clearly defined in a particular context, it is challenging to quantify. We identify four characteristics of the response of a system function to disturbance that relate to “resilience”: (1) degree of return of the function to a reference level; (2) time taken to reach a new quasi-stable state; (3) rate (i.e. gradient) at which the function reaches the new state; (4) cumulative magnitude of the function (i.e. area under the curve) before a new state is reached. We develop metrics to quantify these characteristics based on an analogy with a mechanical spring and damper system. Using the example of the response of a soil function (respiration) to disturbance, we demonstrate that these metrics effectively discriminate key features of the dynamic response. Although any one of these characteristics could define resilience, each may lead to different insights and conclusions. The salient properties of a resilient response must thus be identified for different contexts. Because the temporal resolution of data affects the accurate determination of these metrics, we recommend that at least twelve measurements are made over the temporal range for which the response is expected.

The term “resilience” is one much used in the ecological, complexity and systems literature^1^. However, whilst the concept of resilience is readily understood in a general sense, its specific meaning can be context-dependent and open to wide interpretation^1^,^2^,^3^. Even when clearly defined, resilience is difficult to quantify, making it challenging to compare the resilience of different systems and hence identify different factors that contribute to it. These ambiguities have led to a number of confusing and sometimes conflicting views of resilience in the literature.

Two conceptual definitions of resilience are widely used in systems science, generally referred to as *ecological* resilience and *engineering* resilience^1^. Ecological resilience is based on the concept of Holling^4^ and has been defined as the capacity of a system to tolerate disturbance without changing to an alternative configuration, and is therefore important for maintaining desired ecosystem functions (e.g. ref. 5). In contrast, engineering resilience is based on the concept of Pimm^6^ who defined resilience as the time taken for a system to return to its pre-disturbed, stable “state”. Thus the engineering resilience is a measure of the dynamics of the system in the region of a stable equilibrium. These definitions, however, are in a state of flux in the current literature (e.g. refs 3 and 7). Furthermore, there is confusion around what constitutes a disturbance versus a perturbation. We here define *disturbance* as a sudden shock imposed on the system by a change in conditions external to the system (e.g. a sudden increase in ambient temperature) and *perturbation* as the change in the level of function of a system due to such a disturbance.

Resilience is also a topical issue in soil science as climatic variability and land management practices impose frequent, sudden disturbances to soils that perturb both the state (e.g. microbial configuration, physico-chemical properties) and functions (e.g. carbon cycling, nitrogen cycling, etc.) of a soil. As such, there is an increasing desire to quantify the “resilience” of soil functions to such disturbances in order to identify land management practices that minimise any negative effects of these disturbances. In this context the resilience of soil biological function is often assayed by monitoring the dynamics of a chosen soil function in response to an imposed disturbance^8^,^9^,^10^,^11^,^12^,^13^. Such measurements are then used to identify soil properties and conditions that contribute to resilient responses^8^,^9^,^10^,^11^,^12^,^13^,^14^.

At first glance, the approach of monitoring perturbation in a soil function resembles an assay of the engineering resilience; however, the imposed disturbance may alter the system such that the observed soil function tends towards a new stable equilibrium, rather than returning to the prior equilibrium. In fact, reviews of the behaviour of microbial communities^15^,^16^ have concluded that this is often the case, because microbial composition and community functions rarely return to their prior stable state or level of function after a disturbance. The measured time-series of the response of a soil function to a disturbance thus provides an insight into the short-term dynamics of the soil whether or not it returns to the prior equilibrium, and it is clear that this response pertains to the resilience of the system to the disturbance. To be consistent with this approach, however, the “resilience” should be viewed as a property of the dynamics of the response, rather than a property of the system itself. Given their relatively rapid responses to disturbance, soil microcosms provide an effective experimental resource to advance the understanding of resilience as viewed in this way.

Previous methods have been developed to quantify the resilience of soil functions to disturbance from a time-series of the response (e.g. refs 17, 18, 19, 20, 21). Many of these existing metrics of resilience are derived as indices based on a comparison between measurements of a function in a perturbed and control (non-perturbed) soil^17^,^18^,^19^,^20^. Because it can be technically challenging to capture the dynamic responses of soils at high temporal resolution, existing metrics tend to be based on measurements at nominally key points in time such as (what is assumed to be) the occasion of maximum difference between control and treatment, or the difference between control and treatment at a prescribed end of the assay. If measurements are made over a period of time, however, it is not always clear how to compare the resilience of two or more soils using these metrics, because comparisons must be made between several time series rather than values measured at a particular time. An alternative measure of resilience uses a calculation of the area under a curve defined by a time series of the response to a perturbation^21^. Whilst this quantifies something of the total response in a single number, other aspects of the dynamic response are not represented, including the time taken for the function to reach a new stable equilibrium, or even whether the system reaches such a new stable equilibrium. Metrics that can be interpreted when a time series of the response is available would therefore be useful and would allow more accurate comparison of the dynamics of soil responses to disturbance. In order to do this, we need to consider and clarify what a “resilient response” looks like.

In this paper, we first identify and discuss the key characteristics of a resilient response to a disturbance in a general sense. We then develop metrics for these characteristics using an analogy with a mechanical spring and damper system and demonstrate their use by applying them to data collected using soil microcosms. Specifically, we use time series of soil respiration following disturbance from three perspectives, firstly using synthetic data, secondly via examples from published datasets and thirdly using a newly-derived high temporal resolution dataset. Since the accurate identification of the metrics is sensitive to the temporal resolution of the data, we further consider the number of data points needed to estimate the resilience metrics optimally. We recognise that resilience might be a more holistic property of a soil or system, but in order to make progress we will focus on the dynamics of a single response variable (gross soil respiration) which results from a single disturbance.

## Theory

### Characteristics of a resilient response

**Figure 1:**
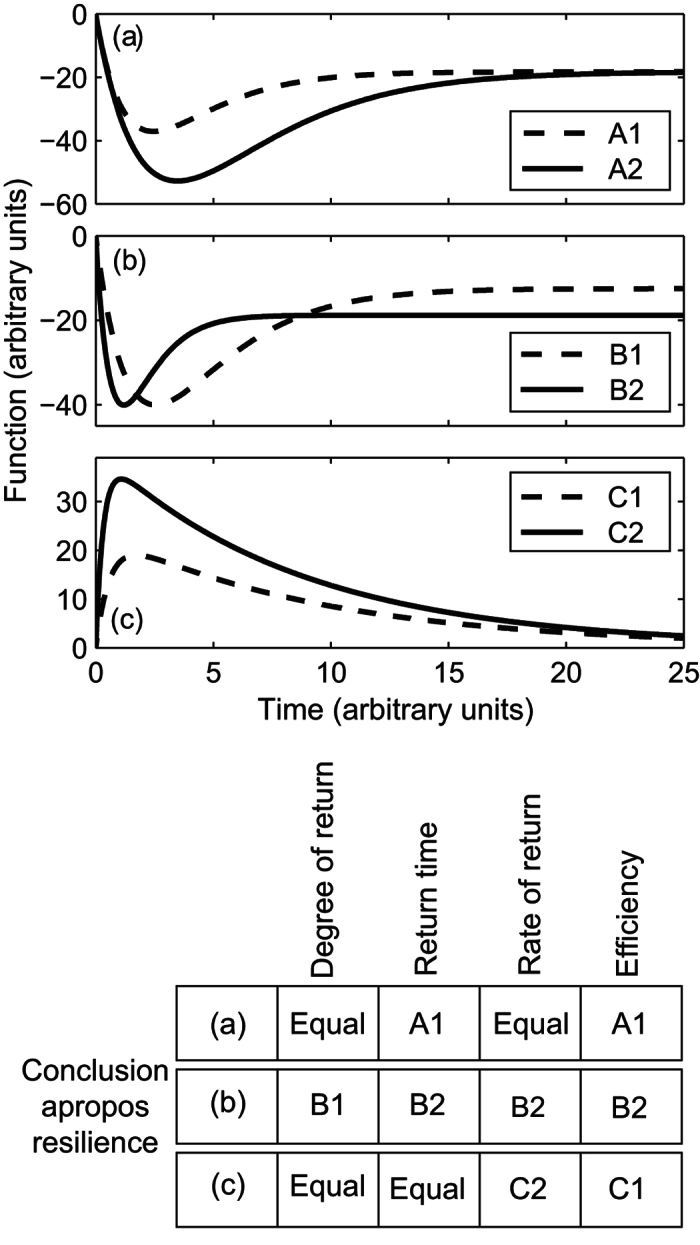
Comparisons of three, hypothetical pairs of responses (**a–c**) to a disturbance and (table inset) the conclusions that can be drawn to identify which of each pair is more resilient when comparing the four resilience characteristics identified in Table 1.

Consider the pairs of responses of a system shown in Fig. 1; within each pair, which response is more resilient? Arguably, this will depend upon which particular characteristics of the response curve are being considered. Here, we propose four key characteristics of the response to a disturbance that relate to different nuances of resilience concepts, *viz*. the degree of return to the original state or level of delivery of a function, the return time, the rate or return, and the “efficiency” (Table 1). The use of each of these different characteristics to identify a resilient response can lead to different conclusions regarding which is the most “resilient”. Using any one of these four characteristics alone (applied qualitatively) to compare the pairs of responses shown in Fig. 1 leads to different conclusions regarding which response is more resilient. For example, Response B2 could be considered more resilient because it returns to a stable equilibrium more quickly than Response B1 (Fig. 1b). However, Response B2 could also be considered less resilient, because Response B1 ultimately returns to a stable equilibrium closer to that of a control sample. Calculation and comparison of resilience indices (e.g. ref. 20) in this hypothetical example at 25 h, would constitute a comparison of the new stable equilibriums observed from the two responses, but it is less clear how the other characteristics could be considered using resilience indices calculated and compared at a particular time. This is especially true for the comparison of B1 and B2 as the response curves intersect repeatedly. Metrics that describe these characteristics, however, would provide a clearer prescription of which feature or features are being considered when quantifying the resilience of a response. Different characteristics may be more relevant in different contexts, yet the use of a different characteristic or characteristics could also lead to different conclusions, and thus clearly requires consideration.

The *degree of return* is frequently suggested as a metric of resilience^2^,^22^,^23^, and is a measure of the extent to which the observed function comes back to a prescribed reference level. This reference level could be the level of function before the disturbance, or the level of a control (unperturbed) sample as, in determining this reference, it may be necessary to account for underlying trends modulated by other factors such as background environmental conditions which both control and perturbed samples may be subjected to. The degree of return also relates to the concept of ecological resilience as, if the system had reached a tipping point, we might expect to observe a change in the functioning. However, as discussed previously, small changes in soil functioning after disturbance are also common^15^. Note that we use the degree of *return* rather than *recovery* because, in some cases, a large value of the degree of return (i.e. it returns to a level of function very different to that of the reference level) could constitute soil recovery, as this new level of function could be more desirable. An example would be in the case of a heavily contaminated soil, which in its contaminated form is functionally moribund (but highly resilient in one sense), and where the desired state is a higher level of function. Thus resilience is, in fact, unhelpful in this remediation context, as it hinders soil recovery^7^.

The *return time* (i.e. the time taken to return to a (quasi) stable level of function) is similar to Pimm’s original definition of engineering resilience which is widely suggested as a second metric of a resilient response (e.g. refs 2, 22). In these definitions the time to return to the prior “state” and, by implication, the prior level of function is sought. However, here we define the return time as the time taken for the system to return to an equilibrium level of function, even if the new equilibrium level is different, and as such, even if an ecological resilience threshold is passed. This characteristic therefore quantifies the length of the transient response period of the observed function.

Although sometimes confused with the return time, the *rate of return* is, in effect, a combined measure of the return time and the magnitude of the perturbation of the function during the transient response. The maximum perturbation (i.e. the magnitude of the peak response) often affects the magnitude of the observed gradient of the response. As such, this measure provides a metric of the rate of change in the function during the transient response period. Notably, using this measure, the applied disturbance must be severe enough to perturb the level of functioning, otherwise no gradient in the response is observed and this characteristic cannot be quantified.

The use of the area under the functional response curve as a resilience characteristic to describe the *efficiency* of the response presupposes that the transient changes in function observed after a perturbation are undesirable. If there is a loss of function, the area under the curve will be negative, which could correspond to the cumulative loss in function when the system is perturbed. However, if the function is increased by the perturbation then the area under the curve would become positive, which could also be deemed an inefficiency if it means that a limited resource is being used more quickly. Using this characteristic, the most resilient response is that where a function is unperturbed by a disturbance. Thus, unlike the rate of return, this characteristic can be quantified when a function does not change in response to a disturbance.

Although the “resistance” (which can be defined as either the immediate or the maximum perturbation in function after a disturbance) is also often considered as a component of resilience, this is not a dynamic characteristic of the response but simply the magnitude of the response at a particular point in time. The resistance, however, affects the rate of return and efficiency characteristics thus these two metrics capture the key effects of the resistance and provide a more reliable measure because they make use of all of the available data, rather than a single point.

Together, the four characteristics proposed here summarise the dynamic response of a soil function to a perturbation. However, these four characteristics are not independent of each other (e.g. the efficiency in terms of the area under the curve will increase if the return time increases or if the rate of return decreases). As such, the combination of these four characteristics into a single value would double-count some aspects of the response. Instead, here we develop and calculate metrics to quantify all four of these characteristic, each of which provides different insights into the dynamics of the response.

## Methods

### Quantifying the resilience characteristics

**Figure 2:**
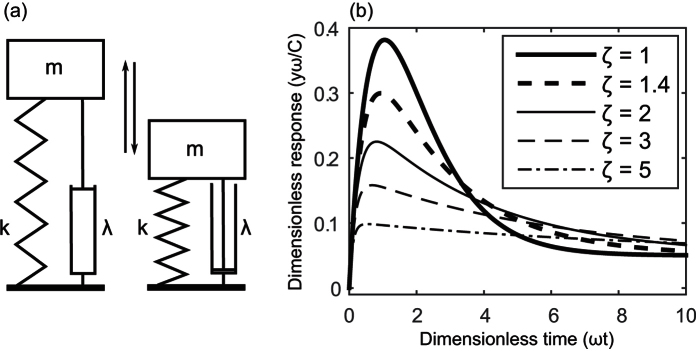
(**a**) Diagram of the mechanical spring damper system, with mass *m*, spring constant *k* and damping constant *λ*, used as an analogy to develop a model of soil function perturbation after a disturbance. (**b**) The dimensionless response of this spring damper system for different damping factors (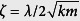), plotted for a new equilibrium position at 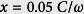. See Supplementary Methods for derivation of formulae.

**Figure 3:**
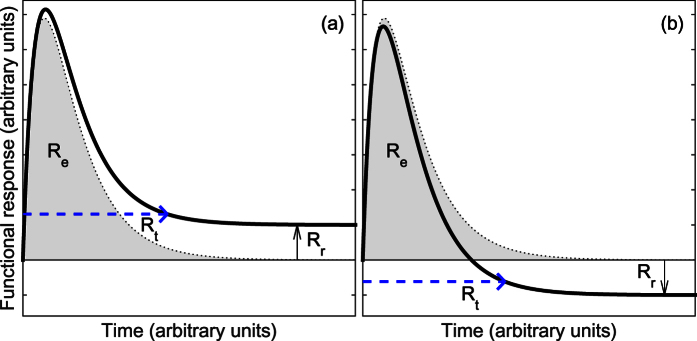
Diagrams to show how the resilience characteristics for degree of return (*R*_*r*_), return time (*R*_*t*_), and efficiency (*R*_*e*_) are quantified from the modelled response curve. *R*_*g*_ is quantified as the average magnitude of the gradient of the curve between 0 and *R*_*t*_.

The identification of these four resilience characteristics provides new clarity in describing the resilience of a system’s functional response to disturbance. There are, however, many ways in which these characteristics could be quantified. Here we propose metrics based on a model derived from an analogy with a mechanical spring and damper system (or, equivalently an electrical analogy of a circuit formed from a resistor, capacitor and inductor). In the mechanical system (Fig. 2a), if the mass is displaced it will return to its equilibrium position in a manner defined by the forces acting on the mass due to the spring and damper. At any point in time, the force imposed by the spring is proportional to the distance between the mass and the equilibrium position and will act to accelerate the mass towards the equilibrium position. The damping force is instead proportional to the velocity and acts to retard the movement of the mass, reducing its momentum. By analogy, as has been suggested previously^24^,^25^, we conceive of tendencies in biological systems such as soil microbial communities to (i) return towards the original state or functional behaviour and (ii) impart resistance to any change. We model these tendencies by assuming the simplest possible form of such a response, i.e. by assuming the system responds as a spring and damper. A differential equation to resolve the effects of these forces can be solved analytically to describe the response of a system after a sudden disturbance (see Supplementary Methods). If we also include a change in the equilibrium position that the system eventually returns to, the response can be described by four parameters:

- *x*–the new stable state of the system
- *C*–the gradient of the response curve at *t* = 0, which relates directly to the magnitude of the sudden, initial perturbation of the system.
- *ω*–the natural frequency, which describes the frequency with which the system would oscillate if no damping occurred.
- *ζ*–the damping factor, which is illustrated in Fig. 3b and describes how the response is damped over time. Notably, when the system is “critically” damped (*ζ* = 1) the system returns to equilibrium in the shortest possible time. As values of the damping factor differ from 1 the return time increases. We consider only “over-damped” solutions, where *ζ* > 1 and the system does not oscillate, corresponding to what was observed from the experimental data.

The response curve defined by the spring and damper analogy can then be fitted to experimental data of a function response over time in order to identify the model parameters (*x, C*, *ω* and *ζ*). The parameters ω and ζ might themselves be considered as metrics of a resilient response if we take the spring-damper metaphor more literally. However, we have not considered this interesting approach further for lack of suitable data. Instead, the fitted response curve is used to quantify the four resilience characteristics as follows:

- *R*_*r*_ = *x*, the degree of return is directly quantified by the model parameter *x* (Fig. 3a).
- The return time (*R*_*t*_) is quantified by *R*_*t*_ = *5 ζ/ω*. In this expression the damping factor, *ζ*, which is characteristic of the temporal response when the response is considered non-dimensionally (Fig. 2b), divided by *ω* in order to convert this into a characteristic time of the response. As what constitutes the “return” of the system is subjective, we use the factor 5 as this corresponds to approximately a 95% return of a critically damped system to a new stable state (Fig. 3a).
- The rate of return (*R*_*g*_) is quantified by calculating the mean value of the magnitude of the gradient between zero and the return time.
- The efficiency characteristic (*R*_*e*_) is quantified as the area under the response curve, assuming that the system returns to its prior state but with all other parameters remaining the same (Fig. 3a). The assumption in this calculation that the system returns to the prior state, removes the difficulty of interpretation that could occur if the response crosses the reference state (Fig. 3b), as in this situation areas below the x-axis will have a negative value and above the axis will be positive and may thus cancel each other out.

These resilience characteristics were estimated, along with their uncertainties following the method presented in the Supplementary Methods which also include a script written in *R* that can be used to fit these metrics. The estimates of the uncertainty do not account for uncertainty in the choice of model structure, thus it is assumed when fitting that the form of the response curve resembles the responses shown in Fig. 2b. Whilst these metrics have been derived by assuming an expected form of the response, the resilience characteristics are more general and could be applied to different types of response using a different model.

### Applying the model to data

First, to test the ability of the metrics to discriminate between responses, we estimated the metrics for the four resilience characteristics for noisy, generated, synthetic data sets based on the three pairs of examples in Fig. 1 (see Supplementary Methods for details of how noise within the synthetic data was modelled). Then, in order to consider both the insights these metrics could bring and practicalities of applying the model to real data, we applied the method to three published experimental data sets from measurements made on soils (Table 2). As measurements of some soil functions can be demanding or expensive, we also used a third newly-generated experimental data set with high temporal resolution to investigate the minimum number of measurement points needed in order to estimate and compare the metrics reliably.

## Experimental Methods

Datasets which reflect the broad biological functioning of the soils in terms of their ability to process a known input of carbon after a disturbance were adopted. Apropos the published data, Kuan *et al*.^12^ and Gregory *et al*.^9^ disturbed 26 and 15 different soils respectively by heating to 40 °C for 18 h. Triplicate measurements of substrate induced respiration (total CO_2_ efflux from 10 g of soil mixed with 100 mg of powdered barley shoots (4.6 mg C g^−1^ soil) over 24 hours at 16 °C) were made at 5 time points post-disturbance (1, 3, 8, 14 and 28 days). Controls (no powdered barley shoot addition) were assayed at the same time points and the measurements expressed as the percentage difference between the perturbed and control responses. Butler *et al*.^26^, disturbed 50 g triplicate samples of soil (3 different textures; sandy loam, clay, and loamy sand) with the addition of 1000 mg kg^−1^ dry mass of the anti-microbial substance triclosan. After 0, 1, 2, 3, 4, 5, 6, 14 days of incubation in the presence of triclosan, 1 g aliquots of soil were used to measure substrate induced respiration (SIR) by adding texture-dependent volumes of 200 mM glucose solution.

High temporal resolution data was obtained by incubating 2 soils (a cambisol used as pasture and a stagnosol supporting a mixed woodland) for 5 days (0.5 g samples mixed with 5 mg of powdered barley shoots so as to provide approximately 2.3 mg C per g soil) and then subjected to one dry:wet cycle (3 days dry in desiccator with silica gel: 5 days rewetted to 45% of gravimetric water-holding capacity). Respiration was measured using an automated respirometer based on conductimetric detection of evolved CO_2_^26^ at six-minute intervals over the 5 day period, and averaged to hourly rates. The respiration data and further information about the two soils are included in the supplementary data.

## Results and Discussion

### Synthetic data

**Figure 4:**
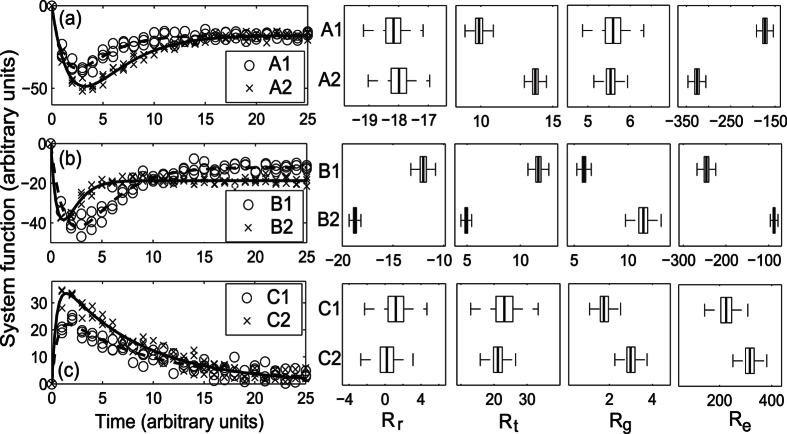
Synthetic data and simulated, fitted responses based on the three pairs of responses shown in Fig. 1 (a–c) along with values of degree of return (*R*_*r*_), return time (*R*_*t*_), rate of return (*R*_*g*_) and efficiency (*R*_*e*_) estimated from these fitted responses. Boxplots show the median (central mark), interquartile range (box) and range (whiskers) with outliers excluded.

When applied to noisy, synthetic data generated from the pairs of examples from Fig. 1, the fitted metrics were able to identify the expected differences in the resilience characteristics in the pairs of responses in most cases (Fig. 4). For example, the metrics showed that, as expected from Fig. 1, Response A1 returned to an equilibrium more quickly than Response A2 (*R*_*t*_, Fig. 4a), but no difference in the degree of return (*R*_*r*_) of the two responses was observed. However, when the difference in the observed values was small, as with the estimates of the efficiency characteristics (*R*_*e*_) for responses C1 and C2 (Fig. 4c), uncertainty in the estimated metrics did not allow clear discrimination between the two responses, even when a difference was expected.

### Heat disturbance

**Figure 5:**
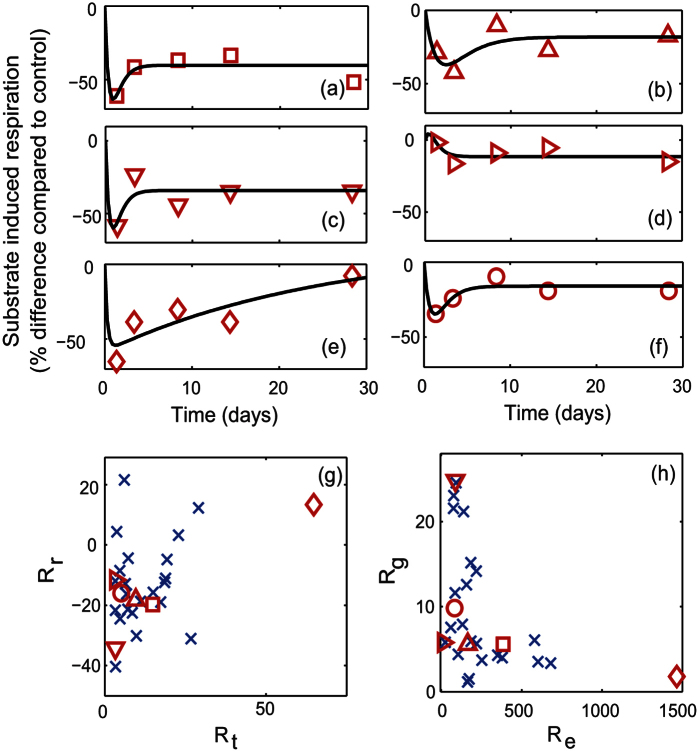
Six indicative examples (**a–f**) of the response of SIR after exposure to heat stress showing the derived resilience metrics (**g,h**) (*R*_*r*_*, R*_*t*_*, R*_*g*_ and *R*_*e*_, Table 1) for the 24 soil samples in which the model identified the degree of return. Symbols in plots (**g,h**) correspond to those in the responses plots (**a–f**), metrics for the responses of other soils are shown as crosses. Confidence intervals for these metrics are large (due to the small number of data points) and are not shown. Different soils are identified as more resilient depending on which characteristic is used to rank the responses. For example, the response in plot (**c**) is identified as the most resilient using the rate of return metric (*R*_*g*_) because the gradient during the response period is large. However, it is one of the least resilient responses in terms of the degree of return (*R*_*r*_) as it returns to a level which is very different to that of the control (i.e. far from zero). The response in plot (**e**) is identified as the least resilient using all the metrics except *R*_*r*_ as it eventually returns to a level similar to that of the control.

When fitted to data from experiments in which a number of soils were exposed to a heat disturbance, the model represented the observed perturbation in induced respiration well in several cases (Fig. 5). However, the model was unable to identify the new stable level in 17 of the 41 soils, resulting in infeasible values of *R*_*r*_. Hence it would be preferable to have more points in the time series to improve the identification of parameters in the model.

When ranked from most to least resilient, the order of the soils varied depending on the resilience characteristic that was used for comparison (Fig. 5). In fact, four different soils were identified as the “most” resilient if this was judged using each characteristic in turn. As such, it is clearly important to consider what features of the response are being sought or understood by the resilience in any given context, in order that analysis of such data sets can compare and contrast the truly salient features.

These new metrics do not contradict earlier work; they tended to rank pairs of soils with the same texture similarly to earlier work^9^. That is to say, where soil from a grassland plot was previously judged to be more resilient than soil from an arable plot of similar texture, for example, our new analysis tended to find the same result. However, the fact that the general order changes with metric illustrates the richness that the approach applied in this paper brings, and the importance of specifying which characteristic of a resilient response is of interest.

### Triclosan disturbance

**Figure 6:**
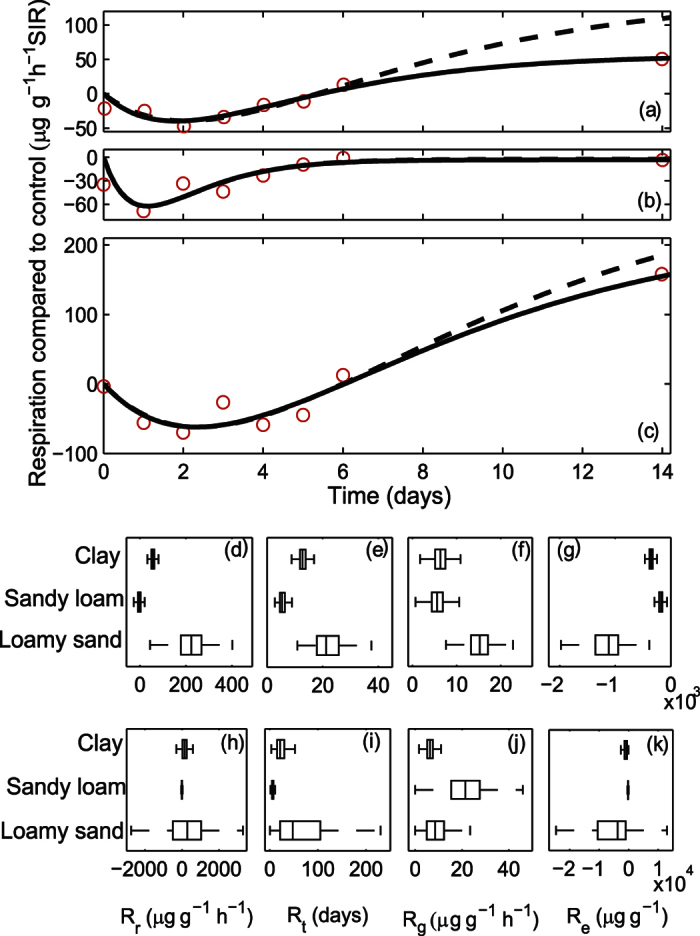
Change in substrate induced respiration of (**a**) clay, (**b**) sandy loam and (**c**) loamy sand soil samples after the addition of 1000 mg triclosan kg^−1^ dry mass showing data (circles) and the modelled response fitted either with (solid lines) or without (dashed lines) the last data point. Note that the two modelled responses are almost identical in the sandy loam. Measurements are the difference in SIR (glucose substrate) between a perturbed and a control sample, with each measurement made destructively. The resilience characteristics (Table 1) identified using the model fitted to all the data (**d–g**) and the model fitted without the last data point (**h–k**). Boxplots show the median (central mark), interquartile range (box) and range (whiskers) with outliers excluded.

Three of the four metrics suggested that SIR in the sandy loam soil was more resilient to the addition of triclosan than in the clay or loamy sand soil (Fig. 6d–g). There was no difference identified between the rate of return (*R*_*g*_) of the soils. The single measurement made 14 days after the addition of triclosan had high leverage (Fig. 6h–k) and particularly affected the values quantifying the degree of return (*R*_*r*_) and return time (*R*_*t*_). For the loamy sand soil, the uncertainty in the degree of return contributed to the large uncertainty in the other estimated metrics. In the previous study^26^ it was noted qualitatively that the sandy loam soil “recovered” more quickly. These new metrics allow this difference to be quantified.

The increase in SIR in the clay and loamy sand samples after 14 days may have occurred because the triclosan was degraded by organisms in the soil. In other words, the context could be interpreted differently, and we might consider that respiration greater than the control sample was the “desirable” response as it may indicate faster degradation of the added triclosan. Thus we should consider whether the specific mechanisms are important in order to quantify the resilience of a response, or whether these should be treated as a black box. Using these metrics, the resilience of a particular function is characterised purely by the dynamic perturbation of the function after the disturbance, irrespective of context and the mechanisms. As such, if the context is important, consideration should be given and the appropriate characteristics identified prior to analysis. For the soils in which the triclosan was degraded microbiologically, there may have been a greater return to the control level beyond 14 days, but this could not have been determined without further measurements.

### Drying and rewetting disturbance

**Figure 7:**
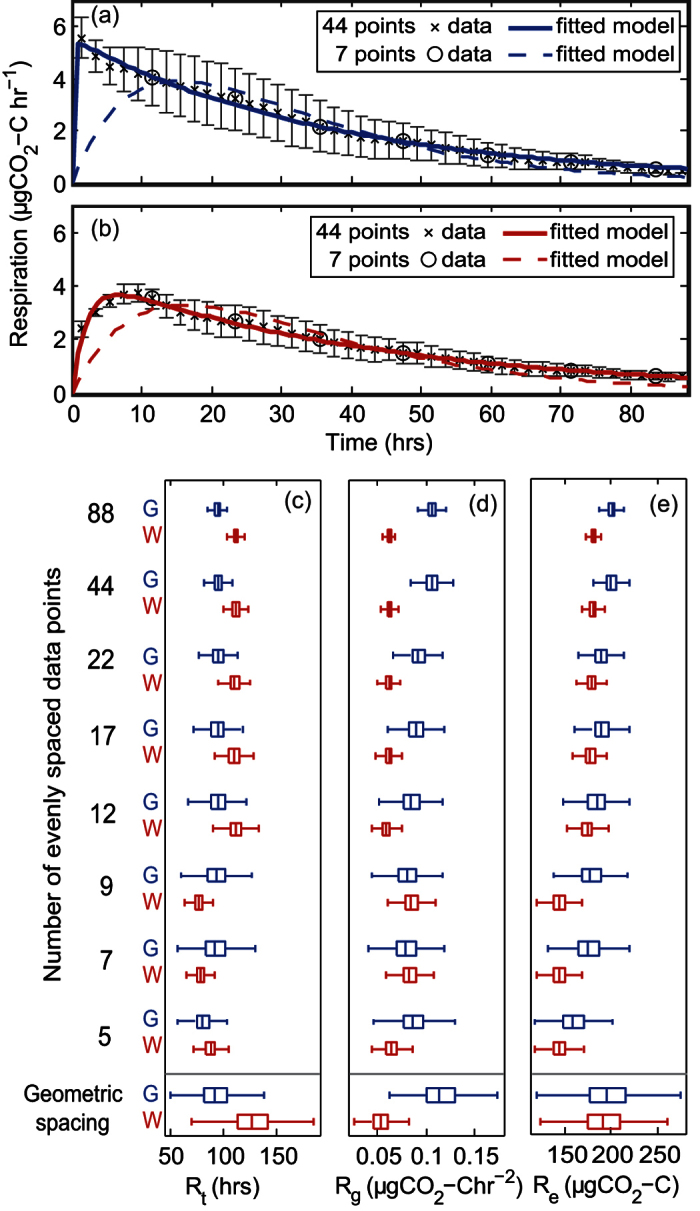
Respiration responses observed after a drying and rewetting disturbance in (**a**) grassland, sandy loam soil and (**b**) woodland, clay loam soil and the corresponding modelled results when fitted to two subsets of the full time series. Error bars indicate ± standard deviation of the data. The estimated (**c**) return time (*R*_*t*_), (**d**) rate of return (*R*_*g*_), and (**e**) efficiency (*R*_*e*_) characteristics for both soils (grassland–*G*, and woodland–*W*) are shown, each estimated using models fitted to subsets of the data with different numbers of data points evenly spaced throughout the measurement period and a geometric spacing of 7 measurements at 1, 2, 4, 8, 16, 32 and 64 hours. Boxplots show the median (central mark), interquartile range (box) and range (whiskers) with outliers excluded, this data is provided in Supplementary Dataset 1. The degree of return was fixed at zero during fitting, as the degree of return was small relative to the peak of the signal and the soils did not return to a stable respiration during the measurement period.

The model represented the form of the response of soil respiration after drying and rewetting of soil (Fig. 7a,b) in the high-resolution experimental data set. The model could not readily identify *R*_*r*_ because a new stable level had not been reached during the measured time period. In this case, as *R*_*r*_ was small compared to the observed peaks in response, the other metrics were estimated by assuming that *R*_*r*_ was equal to zero. Using the resilience metrics to characterise the full high resolution data set of 88 points, it was apparent that the grassland soil returned in a shorter time (Fig. 7c) and at a faster rate (Fig. 7d) than the woodland soil, although the woodland soil had a more efficient response to the disturbance (*R*_*e*_, Fig. 7e).

### Reliability of the metrics

In the triclosan data set (Fig. 6), a repeated measure after 15 days might have helped to ensure that the final level reached in the experimental data was quasi-stable. However, there is also a question about the time scale over which measurements should take place. If the characteristics are estimated by neglecting the final data point at 14 days (Fig. 6h–k) the confidence in the estimated parameters, particularly the degree of return, is reduced and no differences are identified. The time-scale over which measurements should take place is clearly context-dependent, and relates to the relevant time-scale for the response mechanisms. Unsurprisingly, if we want to estimate the degree of return (*R*_*r*_) with confidence, data at a time when the system has actually reached a new stable state is required.

We used the high resolution data set, from the drying and rewetting experiment to investigate systematically how many data are needed to obtain reliable metrics of resilience with our method. Fitting the model using more than 12, evenly-spaced data points yielded consistent estimates of the resilience characteristics (Fig. 7c–e). Increasing the number of data points did, however, improve the confidence in the estimated characteristics and thus increased the ability to discriminate between the two soil responses. Using fewer than 12 data points, the return time (*R*_*t*_) was underestimated compared to using the full data set. Using a geometric spacing of 7 measurement points, in order to sample at higher resolution during the initial period, the median of the estimated characteristics was closer to that estimated using all 88 data points than using 7 data points spaced evenly over time. Nevertheless, the confidence in the characteristics estimated using the geometric series was reduced compared to using 7 evenly space measurements.

### Practical Implications

The choice of which resilience characteristic(s) are used to identify or discriminate resilient responses is context dependent because different features of the dynamics of the response may be considered particularly desirable (or undesirable) in different contexts. As such, it is important that any questions around the resilience of a system are carefully framed; researchers must know what a (non)resilient response will look like for the function measured in relation to the desired outcome.

The degree of return (*R*_*r*_) is likely to be relevant in most contexts because it quantifies the long term ability of the system to continue to function following a disturbance. The other characteristics capture more of the dynamics of the response and may also be of interest in specific contexts. For example, in the above examples from soil science, *R*_*e*_ is likely to be particularly relevant as this provides a cumulative measure of the change in respiration during the time of interest. For the heat and triclosan disturbances, in which the respiration induced by substrate addition can be interpreted as a measure of microbial activity that delivers other functions (e.g. nitrogen cycling) to the surrounding ecosystem, *R*_*e*_ represents the loss in functioning during the dynamic response. For the drying and rewetting disturbance, the increase in respiration after disturbance represents C lost from the system, potentially in an inefficient manner. In the context of the response of soil respiration to a drying and wetting disturbance, the interest may be to minimise the carbon lost (and thus the CO_2_ emissions) from the soil. As such, the efficiency metric (*R*_*e*_) is likely to be of particular interest as it captures the cumulative loss of carbon as a result of the dynamic response to disturbance. The degree of return will also be important as it is also desirable to maintain the microbial community in the soil and so ongoing emissions of CO_2_.

Our characteristics also have potential application in studies of other ecological systems. For example, *R*_*r*_ and *R*_*t*_ could usefully be employed in assessing the effects of overgrazing or predation in both terrestrial^27^ and aquatic systems^28^,^29^, the effect of extreme weather events on populations^30^, and possibly changes in microbiomes due to stress^31^. In these contexts both *R*_*r*_ and *R*_*t*_ could be used by policy makers or businesses for strategic planning or to assess the success of protection or intervention measures. The return rate *R*_*g*_ is likely to be of more interest in studies relating to critical slowing down (the idea that systems respond increasingly slowly as they approach an imminent tipping point). Such gradient measures have previously been used to identify this critical slowing down in both a laboratory experiment using cyanobacteria^32^ and an artificially manipulated freshwater lake food web^33^.

## Conclusions

The metrics proposed here quantify the complete, dynamic range of resilience characteristics (Table 1) of a response to disturbance. With these characteristics and associated metrics, it is possible to articulate the systematic differences in behaviour of a system function that pertain to resilience following a disturbance. For example, if one system returns to a level of function closer to the reference level, whereas another returns to a new equilibrium more quickly, there may be an ambiguity about which response is the more resilient. We quantify such behaviour based on these four meaningful metrics. Their discriminatory power thus provides an opportunity to analyse the response of systems to disturbance in new ways and resolve aspects of otherwise complex-seeming interactions that contribute to different aspects of a “resilient” response.

If sufficient resources are available, more than about 12 temporal data measurements should be made in order to estimate the suite of resilience metrics reliably. If the return time, *R*_*t*_, is thought important, care must be taken to ensure that the experimental response is followed for long enough to determine this.

## Additional Information

**How to cite this article**: Todman, L. C. *et al*. Defining and quantifying the resilience of responses to disturbance: a conceptual and modelling approach from soil science. *Sci. Rep.* **6**, 28426; doi: 10.1038/srep28426 (2016).

## Supplementary Material

Supplementary Information

Supplementary Information

## Figures and Tables

**Table 1:** Summary of four characteristics associated with responses to disturbance enabling description of a resilient response.

Characteristic^1^	Symbol^2^	A more resilient soil response…	Units^3^
(i) Degree of return	*R*_*r*_	Returns to a stable level of function closer to a reference level (e.g. the initial level of function or level of a control sample)	U
(ii) Return time-The time taken to reach the new stable level of function	*R*_*t*_	Reaches the stable level of function more quickly	T
(iii) Rate of return-The rate at which the response tends towards the stable level of function (i.e. related to the gradient of the return period)	*R*_*g*_	Has a steeper gradient during return	UT^−1^
(iv) Efficiency	*R*_*e*_	Has a smaller area under the response curve i.e. is away from the reference level for less time in total	UT

^1^These characteristics could be applied to any time series that measures a change in a variable after a disturbance. Here we assume that the variable is an observed function of the system, but the variable could also represent a state of the system (e.g. microbial biomass carbon) if this were observed.

^2^Formal definition and derivation of these are presented in Supplementary Methods.

^3^Where U denotes the units of the observed function and T denotes a unit of time appropriate to the observed time frame of the response.

**Table 2:** Summary of the experimental data used as examples to assess the resilience characteristics identified in Table 1.

Disturbance	Observed soil function	No. of soils tested	No. of temporal data points	Source
Heating at 40 °C for 18 h	Substrate (barley powder) induced respiration	41	5	(9, 12)
Addition of triclosan (1000 mg kg ^−1^ dry mass)	Substrate (glucose) induced respiration	3	7	26
Drying for 3 days then rewetting to 45% WHC	Basal respiration	2	88	This paper
