# Supplementary material for: Defining and quantifying the resilience of responses to disturbance: a conceptual and modelling approach from soil science
Source: Sci Rep. 2016 Jun 22;6:28426. doi: 10.1038/srep28426 (PMC4916505; doi:10.1038/srep28426)
Supplement: Supplementary Information [file srep28426-s1.pdf]

Supplementary methods

This file contains the supplementary methods to support the manuscript, '*Defining and quantifying the resilience of responses to disturbance: a conceptual and modelling approach from soil science'*, submitted to Nature Scientific Reports

Authors: LC Todman, FC Fraser, R Corstanje, LK Deeks, JA Harris, M Pawlett, K Ritz, AP Whitmore

Model development

Following this analogy of a mechanical spring and damper system and applying Newton's second law of motion, the dynamics of the response to disturbance are described by the ordinary differential equation:

$$m\frac{d^2y}{dt^2} + \lambda\frac{dy}{dt} + ky = kxH(t) \tag{S1}$$

where y (m) is the displacement, m (kg) is the mass, λ is the damping constant (which when multiplied by the velocity gives the damping force), k is the spring constant (which when multiplied by displacement gives the force from the spring), x (m) is the final displacement (which is used in order to represent the expected small changes in soil functioning after a perturbation) and H is a unit Heaviside step function. To eliminate one parameter this can be written as:

$$\frac{1}{\omega^2}\frac{d^2y}{dt^2} + \frac{2\zeta}{\omega}\frac{dy}{dt} + y = xH(t) \tag{S2}$$

where $\omega = \sqrt{k/m}$ (s^{-1}) is the natural frequency (which is the frequency at which the system would oscillate if no damping force were applied) and $\zeta = \lambda/2\sqrt{km}$ is the damping factor. Solving this subject to the initial conditions $\frac{dy}{dt} = C, y = 0$ at $t = 0$ yields:

$$y(t) = \frac{1}{2}\frac{2x\omega^2B + (\omega^2x - AC - BC)e^{-(A+B)t}(A - B) + (-\omega^2x + AC - BC)e^{(-A+B)t}(A + B)}{B(A^2 - B^2)} \quad \zeta > 1 \tag{S3}$$

where $A = \zeta\omega$ and $B = \omega\sqrt{\zeta^2 - 1}$.

Note that for values of damping factor lower than one the mass would oscillate before returning to equilibrium, but we do not consider this response here. The initial condition $\frac{dy}{dt} = C$ implies that an impulse disturbance is imposed on the system at time 0, such that the gradient of the function changes instantaneously at this point. The disturbance to the system is thus represented by a combination of this impulse and the step change represented in equation S2, both of which are considered to occur simultaneously.

Metrics of the resilience characteristics are quantified from this fitted model. As such, the degreee of return is defined as:

$$R_r = x \tag{S4}$$

In some contexts it may be desirable to calculate $|x|$, if it is only a change in value from the reference level that is important rather than the absolute value.

The damping factor (ζ) provides an indicator of the temporal response (on a dimensionless scale) after perturbation. $\zeta = 1$ (critical damping) corresponds to the quickest return to new equilibrium

and increasingly larger values correspond to longer times for return to equilibrium. When applied to data, the peak response may occur at different times (e.g. and the damping factor should be scaled by the frequency parameter, ω. We therefore quantify the return time characteristic as:

$$R_t = 5\frac{\zeta}{\omega} \tag{S5}$$

Whilst the expression ζ/ω is a characteristic time associated with Equation 1, the multiplication by a factor 5 is used so that this time corresponds to approximately 95% return of the signal from to the largest perturbation (peak height) to the final return point (degree of return) for a dimensionless, critically damped system with $R_r = 0$. Comparisons in the characteristic time identified for different soils could be made without using this factor, but we use this 'return time' to develop metrics for the other resilience characteristics.

An alternative to scaling the damping factor relative to the frequency parameter would be to scale relative to the time to peak which can be derived by finding the point at which the gradient of the curve is equal to zero. However, this would increase the sensitivity of results to the accurate estimation of the time to peak, which is likely to require higher resolution of data around the expected time of this peak.

The return rate is quantified by calculating the average magnitude of the gradient during the response period:

$$R_g = \frac{\int_0^{R_t}\left|\frac{dy}{dt}\right|dt}{R_t} \tag{S6}$$

Which can be solved numerically or analytically by identifying a turning point (if any) at which the gradient equals zero.

As the degree of return is already quantified by R_r, here R_e is calculated as the area under the response curve assuming that it returns to it's initial state (i.e. $x = 0$). If the degree of return was also an important component of the 'efficiency' the two resilience characteristics could be considered to give a more complete description of the response. Note that we calculate the area within the time range from zero to twice the return time as beyond this value the reponse tends to zero, thus:

$$R_e = \int_0^{2R_t}\frac{1}{2}\frac{(AC-BC)e^{(-A+B)t}(A+B)-(AC+BC)e^{-(A+B)t}(A-B)}{B(A^2-B^2)}dt \tag{S7}$$

In addition to reducing the sensitivity to the time range, this formulation removes the difficulty of interpretation that could occur if the response crosses the reference state, as in this situation areas

below the x-axis will have a negative value and above the axis will be positive and may thus cancel each other out.

If the system is assumed to return eventually to its initial state (i.e. x=0 in S1), a simpler set of solutions is found with an overdamped, critically damped and underdamped responses given respectively as

$$\begin{aligned} y(t) &= \frac{C}{B} e^{-At} \sinh(Bt), \\ y(t) &= Cte^{-At} \\ y(t) &= \frac{C}{iB} e^{-At} \sin(iBt) \end{aligned} \tag{S8}$$

Parameter R_r was large in almost all of the measured data and thus justifies the more complex formulation (S3).

Model fitting and estimation of resilience characteristics

The model (Eq. 1) was fitted to data in order to estimate the four parameters (x, C, ω and ζ). This fitting was conducted in two stages. First, the parameters were optimised within a constrained range of parameters using the MATLAB function *lsqcurvefit* which uses iterative least squares. This optimisation was started from multiple points (50-100) within the constrained region, so that a global rather than a local optimimum was found. The parameter ω was constrained such that $1/\omega$ was larger than half the measurement interval, or half of the time to the first measurement. This was done so that the fitted parameter values did not simulate an immediate peak in the simulated response. Whilst low resolution data could mean that a peak of this kind does in fact occur more than half of the time before the initial measurement is made, this cannot be identified by the model without additional data. The optimum parameters from the first, constrained routine were then used to initialise a further, unconstrained fitting algorithm (*nlfit*) in order to estimate the parameters and the covariance matrix of these parameters. If the first stage of the optimisation process identified that the response was likely to be critically damped (i.e. $\zeta = 1$). This parameter was fixed and other three parameters were optimised in the second stage. This improved the convergence of the model.

The parameter estimates and the covariance matrices were then used in a Monte Carlo simulation of 10000 points in order to estimate the probability density function of the four resilience characteristics calculated using equations A1.4-A1.7. This error propagation simulation was performed in @Risk software. In the Monte Carlo simulation, the parameter values for x and C were

each sampled from a normal distribution. Values of the parameters ω and ζ, which have constraints at 0 and 1 respectively, were sampled from lognormal distributions. The correlation between the four parameters was accounted for during sampling. The median, interquartile range and the range (excluding outliers) of each of these simulated populations were then calculated and represented estimates of the different characteristics.

Synthetic data generation

Synthetic data was first generated using equation A1.1 by adding two types of noise to the signal. The first component of the noise was assumed to occur in the four model parameters (ξ_x, ξ_C , ξ_ω and ξ_ζ), simulating a time series of replicates from which repeated measures were taken. A second term (ε) represented white noise in the data and was sampled from a normal distribution with $\mu = 0, \sigma = 2$. Each synthetic time series (y_{synth}) was calculated using the four parameters (x, C, ω and ζ) and the error terms at a series of time points, t, using:

$$y_{synth} = y\left(x + \xi_x, C + \xi_C, \omega + \xi_\omega, \zeta + \xi_\zeta, t\right) + \varepsilon(t)$$

Data was simulated for three replicates (i.e. three values of the parameter errors).

Example of program to calculate metrics

The following script was written in *R*, version i386 3.2.1 for Windows

```
######################### FUNCTION DEFINITIONS #########################
SpringDamperEquation <- function(t, x, C, omega, zeta){
  # function calculates the displacement 'y' at times 't' using the model parameters 'x' (degree of return),
  # 'C' (initial gradient), 'omega' (natural frequency) and 'zeta' (damping factor)

  # calculate useful values to simplify later working
  A<-zeta*omega;
  B<-omega*sqrt(zeta^2-1);

  # calculate the response
  if (B==0){
    y=(x*omega^2+(-A*omega^2*t*x+A^2*C*t-omega^2*x)*exp(-t*A))/A^2
  }
  else {
    y=(1/2)*(2*x*omega^2*B+(omega^2*x-A*C-B*C)*exp(-(A+B)*t)*(A-B)+(-omega^2*x+A*C-B*C)*exp((-A+B)*t)*(A+B))/(B*(A^2-B^2))
  }

  return(y)
}

MinFun <- function(par,data){
  # objective function for optimisation routine
  # calculates the sum of squares error between the simulated and observed data, using the model parameters and observed data
  # data input has a column of measurement times followed by a second column of observed values at these times

  t<-data[,1] # time is first column of data
  obs<-data[,2] # observed data is second column of data

  # extract parameters
```

```
  x<-par[1]
  C<-par[2]
  omega<-par[3]
  zeta<-par[4]

  # simulate response
  sim<-SpringDamperEquation(t, x, C, omega, zeta)

  # calculate sum of squared difference between simulated and observed results
  lse<-sum((obs-sim)^2)

  return(lse)

}

CalculateCharacteristics <- function(x,C,omega,zeta){
  # function calculates the resilience characteristics degree of return (Rr, RC[1]), return time (Rt, RC[2]), return rate (Rg, RC[3]) and
  # efficiency (Re, RC[4]) based on the fitted model parameters x,C,omega and zeta

  RC=matrix(runif(4,1)) # initialise array

  # degree of return
  RC[1]<-x

  # return time
  RC[2]<-5*zeta/omega

  # calculate useful values to simplify later working to calculate gradient and efficiency
  A=zeta*omega;
  B=omega*sqrt(zeta^2-1);


  # time to peak
  tpeak = (1/2)*log((-omega^2*x+A*C+B*C)/(-omega^2*x+A*C-B*C))/B

  # calculate function value at times 0, tpeak and Rt
  y0 = SpringDamperEquation(0, x, C, omega, zeta)
  ypeak = SpringDamperEquation(tpeak, x, C, omega, zeta)
  yRt = SpringDamperEquation(RC[2], x, C, omega, zeta)

  # calculate average gradient
  RC[3]=(abs(ypeak-y0)+abs(yRt-ypeak))/RC[2]


  # calculate integral of model at times 0 and 2*Rt
  t=2*RC[2]
  Area2<-(1/2)*((-A*C-B*C)*(A-B)*exp(-(A+B)*t)/(-A-B)+(A*C-B*C)*(A+B)*exp((-A+B)*t)/(-A+B))/(B*(A^2-B^2))
  t=0
  Area1<-(1/2)*((-A*C-B*C)*(A-B)*exp(-(A+B)*t)/(-A-B)+(A*C-B*C)*(A+B)*exp((-A+B)*t)/(-A+B))/(B*(A^2-B^2))

  # calculate efficiency
  RC[4]=Area2-Area1

  return(RC)
}

######################### MAIN PROGRAM #########################
# read in and format data
# note that this text file is a copy of the first four columns of the supplementary data file for the woodland soil
DataTemp<-read.table("Woodland.txt", header = TRUE, sep = "\t")

xData=c(DataTemp[,1],DataTemp[,1],DataTemp[,1])
yData=c(DataTemp[,2],DataTemp[,3],DataTemp[,4])

# ObsData contains a column of the time points at which measurements were made and a second column of the measured values
ObsData=cbind(c(xData),c(yData))

# plot the synthetic data
plot(ObsData[,1],ObsData[,2])

# set lower and upper bounds for parameter search range
```

```
Lbound=c(0.00001,1,0.01,1.1) # lower bound for [x, C, omega, zeta]
Ubound=c(20,200,0.5,10) # upper bound for [x, C, omega, zeta]

nPar=4 # number of parameters
# initialise search from the middle of the parameter space
p0=matrix(runif(nPar,1))
for (i in 1:nPar){
  p0[i]=(Ubound[i]+Lbound[i])/2
}

# use R function optim to optimise results
OptimisationResult <- optim(par=p0, MinFun, data=ObsData,method = c("L-BFGS-B"),lower = Lbound, upper = Ubound, hessian = TRUE)

# extract the optimised parameters
ParsOpt<-OptimisationResult$par

# calculate and plot the model using the simulated parameters
tmax=100 #range over which to plot simulated result
tplot<-seq(0,tmax,length=200)
ySim<-SpringDamperEquation(tplot,ParsOpt[1],ParsOpt[2],ParsOpt[3],ParsOpt[4])
lines(tplot,ySim,col="red")

# calculate the resilience characteristics based on these parameters
RC<-CalculateCharacteristics(ParsOpt[1],ParsOpt[2],ParsOpt[3],ParsOpt[4])

# display result on screen
string<-c('Rr','Rt','Rg','Re')
ForDisplay<-cbind(string,RC)
print(ForDisplay)
```
